# Immune response in breastmilk of Black women to SARS-CoV-2 infection and vaccination against COVID-19

**DOI:** 10.3389/fnut.2025.1703784

**Published:** 2026-01-12

**Authors:** Maeve O. Smith, Jessica Price, Ryan Baker, Halle Neely, Dominique Alfandari, Kimarie Bugg, Lindiwe Sibeko, Kathleen F. Arcaro

**Affiliations:** 1 Department of Veterinary and Animal Sciences, University of Massachusetts, Amherst, MA, United States; 2 Reaching Our Sisters Everywhere Inc., Lithonia, GA, United States; 3 Department of Nutrition, University of Massachusetts, Amherst, MA, United States

**Keywords:** Black women, breastfeeding, breastmilk, human milk, lactation, mammary epithelium permeability, SARS-CoV-2

## Abstract

**Introduction:**

In the United States, Black lactating women are underrepresented in health-related studies. This underrepresentation is a concern when interpreting results from studies of the immune response to SARS-CoV-2 in breastmilk because we know that individuals vary greatly in their response to both infection and vaccination. Additionally, few studies of the immune response in human milk include analysis of mammary epithelium permeability, despite the knowledge that elevated permeability can alter constituents in milk. To address these gaps, we enrolled local Black breastfeeding mothers during a 3-day breastfeeding conference in New Orleans to assess the immune response in milk to infection or vaccination with SARS-CoV-2.

**Materials and methods:**

3 weeks prior to the ROSE Black Breastfeeding & Birth Justice Summit of 25–27 August 2022, we advertised for “*Black breastfeeding moms to participate in a health study of the special benefits of breastmilk during COVID-19*.” Consented participants (*n* = 16) received kits with instructions to collect bilateral milk samples, dried blood spots (DBS), and saliva. Concentrations of anti-SARS-CoV-2 antibodies against both the Wuhan and Omicron variants were determined in milk, DBS, and saliva using the ELISA test. The concentration of a panel of cytokines was determined in milk, and the permeability of the mammary gland was assessed.

**Results:**

Among the 16 lactating women who provided samples, 8 had a positive COVID-19 test within the previous 19 months, and 12 had received an mRNA-based COVID-19 vaccine within the previous 16 months. Milk and maternal blood spots from all participants were positive for all anti-SARS-CoV-2 antibody classes tested, while only a subset of saliva samples were positive for all anti-SARS-CoV-2 antibody classes. A significant correlation was found between mammary epithelium permeability and concentrations of IL-1β, IL-6, and IL-8 cytokines.

**Conclusion:**

Results from this small pilot study supported the need to include a diverse population in breastmilk studies, as the immune response in milk varied greatly among individuals. Future studies assessing the response to infections and vaccinations in lactating women should include analysis of milk from both breasts, as well as assessment of mammary epithelium permeability.

## Introduction

We and others have observed substantial variability in the immune response present in human milk from women either infected with SARS-CoV-2 or vaccinated against COVID-19 (1–4). For example, milk immunoglobulin (Ig) A antibodies against the receptor binding domain (anti-RBD IgA) varied 10-fold among women with COVID-19 and were not explained by time since first positive test (2, 3). Similarly, the capacity of milk to neutralize variants of the SARS-CoV-2 complex varied greatly among women after mRNA vaccination (4). The wide range in immune responses present in milk from individual women has important implications for the health of the breastfeeding infant (2) and highlights the need for all groups to be represented in health research. In the United States, Black women have been underrepresented in most studies of human milk (5), including many (1–4, 6–11), but not all studies (12) conducted during COVID-19.

Underrepresentation of Black women in human milk research in the United States has recently been addressed by Asiodu and colleagues (5). They conducted a survey of 104 Black pregnant and postpartum people and found that while only 8% had ever been asked to participate in a study of human milk, 59% indicated that they would participate and donate milk if asked. The authors concluded that targeted recruitment with interventions or questions informed by respected community members is needed to enhance the diversity of participants in studies of human milk. In addition, it is important to acknowledge past injustices, address systemic racism, and have continued investment in community-based organizations (13, 14).

Approaches to recruiting diverse populations, such as attending community and state-sponsored events, advertising on social media, requesting healthcare provider referrals, and sending mass emails, have been successful in enrolling groups traditionally underrepresented in health research (15–18). However, there remains a need for novel recruitment strategies formed through partnerships built on mutual trust and respect between researchers and advocacy organizations focused on eliminating health inequities.

Reaching Our Sisters Everywhere Inc. (ROSE) was established in 2011 “*to encourage Black mothers to embrace breastfeeding as a cultural and social norm*” (19). By working with community members on national initiatives, ROSE has been at the forefront of changing the breastfeeding landscape in the United States (19–23). ROSE has helped reduce the barriers Black women face regarding breastfeeding, such as systemic racism, lack of representation among hospital and lactation staff, and less access to educational materials and resources that could support lactation goals (22). Every year, ROSE holds a summit that physicians, researchers, lactation consultants, and families can attend to obtain the latest findings on breastfeeding research and gain strategies to help promote breastfeeding in their communities. Thus, collaborations with ROSE may provide one approach to obtaining diverse populations in studies of lactation and human milk.

Both the humoral and cell-mediated responses against SARS-CoV-2 in milk are important for the health of breastfed infants. Most studies have focused on the humoral response, measuring anti-RBD IgA and IgG in the milk of women either vaccinated or infected after pregnancy. While it is generally accepted that these antibodies in milk only infrequently enter infant circulation (7, 24), they are present in the infant gut (2, 4), where they provide protection, as IgA mediates an early neutralizing response against SARS-CoV-2 at mucosal surfaces (25, 26). Studies of cell-mediated responses reveal distinct memory T cell populations and elevated cytokines in the milk of women either infected with or vaccinated against SARS-CoV-2 after pregnancy (2, 4, 27), potentially providing protective passive immunity (28, 29) and promoting the development of the infant’s immune system (30).

Most analyses of the immune response to SARS-CoV-2 in human milk have not considered laterality (6–8, 10, 12, 31), which is acceptable as bilateral evaluation of milk samples shows that the immune response to SARS-CoV-2 is generally similar across breasts (1, 2, 4). However, local infection, milk stasis, and elevated mammary epithelium permeability (MEP) can significantly impact the immune factors in milk, and we previously reported significant differences in antibody and cytokine concentrations between the left and right breasts that correlated with MEP in the milk of a woman with SARS-CoV-2 (2). Thus, separate collection and analysis of milk from each breast can provide the opportunity to further our understanding of the contribution of local factors on the immune response present in milk, and also may be relevant to infant feeding preferences and maternal breast health.

Comparisons of the immune responses present in milk and blood (either maternal or infant) have been informative during COVID-19 (2, 4, 7, 24, 27, 32); however, collecting blood greatly limits the study design and participation. Collecting fresh blood requires trained researchers, is costly, and invasive. Dried blood spots (DBS) remove some of the burden as they can be collected by the participant at their home, are inexpensive, and less invasive. However, the finger prick is not tolerated by everyone, and many parents may not want to use a lancet on their child. Several studies have shown a correlation between salivary and circulating IgG for SARS-CoV-2 (33–35). But the relationship between milk and maternal salivary levels remains to be determined.

In this pilot study, we used a novel approach to recruit Black lactating parents for a study of the immune response to SARS-CoV-2. We assessed bilateral milk samples for both humoral and cell-mediated responses, as well as mammary gland permeability. Finally, we compared the antibody concentration in milk with that obtained from DBS and saliva.

## Materials and methods

### Designing the study

Leadership at ROSE identified the major questions of interest to their community as follows: (1) Are antibodies protective against SARS-CoV-2 present in breastmilk and maternal blood? (2) For how long after maternal infection or vaccination is the protective response present in milk? Researchers at the University of Massachusetts (UMass) designed the sample collection protocol that included bilateral milk, blood spots, and saliva. Colleagues at ROSE designed the recruitment flyer and approved all study material.

### Recruitment of participants and sample collection

The study was approved by the Institutional Review Board at the University of Massachusetts (UMass) and was promoted on both the ROSE website and through paid Facebook advertisements in New Orleans (Supplementary Figure 1). Advertisements started 3 weeks before the ROSE—Black Breastfeeding & Birth Justice Summit on 25–27 August 2022 at the New Orleans Marriott Warehouse Arts District. Potential participants accessed a form through a QR code and provided contact information. Participants received a consent form through REDCap and were offered the opportunity to discuss the research with a member of the study team. Consented participants received a sample collection kit delivered through the United States Postal Service (USPS). The collection kit included supplies and instructions for collecting bilateral milk samples, dried blood spots (DSBs), and saliva. Participants were also asked to provide demographic and health history information *via* a HIPAA-compliant REDCap questionnaire and to deliver their biological samples to the conference center. Participants were compensated for their time.

### Sample processing

All samples were stored at −20 °C at the conference center and transferred on ice to the laboratory at UMass. Upon arrival at the laboratory, the milk was thawed, and 2-mL aliquots from the left breast, the right breast, and a combined sample with equal volumes from the left and right breasts were prepared and frozen at −20 °C until analyzed. For all analyses of milk, aliquots were thawed, centrifuged at 3220 *g* for 3 min, and the fat layer was removed *via* aspiration, leaving the whey fraction for analysis. DBSs and saliva samples for the ELISAs were prepared using previously described methods (33).

### Enzyme-linked immunosorbent assay to assess anti-RBD antibodies

ELISAs were designed to detect either anti-Wuhan receptor binding domain (RBD) antibodies or anti-Omicron (BA.4/BA.5) RBD antibodies. Both anti-SARS-CoV-2 immunoglobulin (Ig)G and IgA antibodies were assessed in milk and blood samples. Only IgG antibodies were measured in blood, as IgG is the predominant antibody class in blood (36). ELISAs were conducted with methods previously validated at UMass for milk (1) and DBS and saliva (33), with the following adjustment. A milk sample comprising equal volumes from the left and right breasts was used for analysis of antibodies. The combined milk samples were serially diluted 1:4 three times, providing four dilutions used in the assay. The initial DBS samples were serially diluted 1:4 six times, providing seven dilutions for use in the assay. The undiluted saliva samples were used in the assay along with a saliva sample that had been diluted 1:2.

Antibody concentrations were interpolated using Prism 10 software and standard curves with known concentrations of anti-SARS-CoV-2-Wuhan-Hu-1 IgG and IgA (33). All samples were tested in technical duplicates and only samples with coefficient of variations (CVs) below 10% were included. At the time of data collection, an anti-SARS-CoV-2-Omicron standard was not available, so the same standard curve was used for anti-RBD-Omicron and Wuhan antibody concentrations.

### Sodium to potassium ratio in human milk to assess mammary epithelium permeability

To assess mammary epithelium permeability, the ratio of sodium (Na) ions to potassium (K) ions was determined in the whey fraction of each milk sample using ion-specific electron probes (Medica Easylyte Na: K Analyzer), as previously described (37).

### Detection of cytokines in human milk

The Mesoscale Discovery 10-plex human V-PLEX Proinflammatory Panel 1 (cat. # K15049D-2) multiplex assay was used to measure cytokine levels in the whey fraction of bilateral milk samples according to the manufacturer’s instructions. Levels of 10 cytokines were assessed: Interleukin (IL)-2, IL-4, IL-6, IL-8, IL-10, IL-12p70, IL-1β, interferon-gamma (IFN-*γ*), and tumor necrosis factor-alpha (TNF-*α*). Samples and standards were added in technical duplicates.

### Statistical analysis

GraphPad Prism 10 was used to compare groups (*t*-tests) and assess relationships (Pearson R) in all data sets. All *t*-tests were two-tailed, and a *p* < 0.05 was considered significant. Significance was adjusted with a Bonferroni correction when comparing the four antibodies in milk and in blood. Samples with levels of anti-RBD IgG and IgA antibodies three times above the corresponding background were defined as positive.

## Results

### Participant recruitment and demographics

Within a 15-day period (2–19 August 2022), 22 people responded to the advertisement. Twelve of these signed the consent document and were sent collection kits *via* USPS. An additional six people contacted the research staff at the conference, and five enrolled and received kits. Of these five, four were attendees at the conference, and one was a walk-in having heard about the study through a friend. Of the total 17 consented participants, 16 provided samples and questionnaire data. Four participants requested that their samples be picked up at their homes. Research personnel attending the conference used a taxi service to collect the samples at the homes of these four participants.

Demographic data for the 16 lactating participants are shown in Table 1. All participants self-identified as Black, with two also identifying as Asian. Half of the participants had a prior COVID-19 diagnosis, and 75% of participants had received immunization against COVID-19. Infant age ranged from 1 to 28 months. Every mother in this study had a singleton birth, with most of these births being vaginal deliveries. Five participants were nursing their first child.

**Table 1:** Demographic information (*N* = 16).

Characteristic	n* (%)
Age (years)	
20–24	4 (26.67)
25–29	4 (26.67)
30–34	3 (20)
34–39	4 (26.67)
Self-identification	
Black	16 (100)
Asian	2 (13.33)
Vaccinated against SARS-CoV-2	
No	4 (26.67)
Yes	11 (73.33)
Number of COVID-19 infections	
0	8 (53.33)
1	7 (46.67)
Age at first live birth (years)	
15–19	4 (26.67)
20–24	5 (33.33)
25–29	3 (20)
30–34	3 (20)
Pregnancies resulting in live births	
1	5 (33.33)
2	4 (26.67)
3	4 (26.67)
4	2 (13.33)
Age of infant (months)	
1–6	6 (40)
6–12	6 (40)
13+	3 (20)
Birth method	
Vaginal	13 (86.67)
Cesarean	2 (13.33)
Sex of infant	
Female	8 (53.33)
Male	7 (46.67)
Breastfeeding	
First child	5 (33.33)
Second child	4 (26.67)
Not specified	6 (40)

*Some data are missing for one participant.

### Levels of antibodies vary among tissue types and individuals

**Figure 1:**
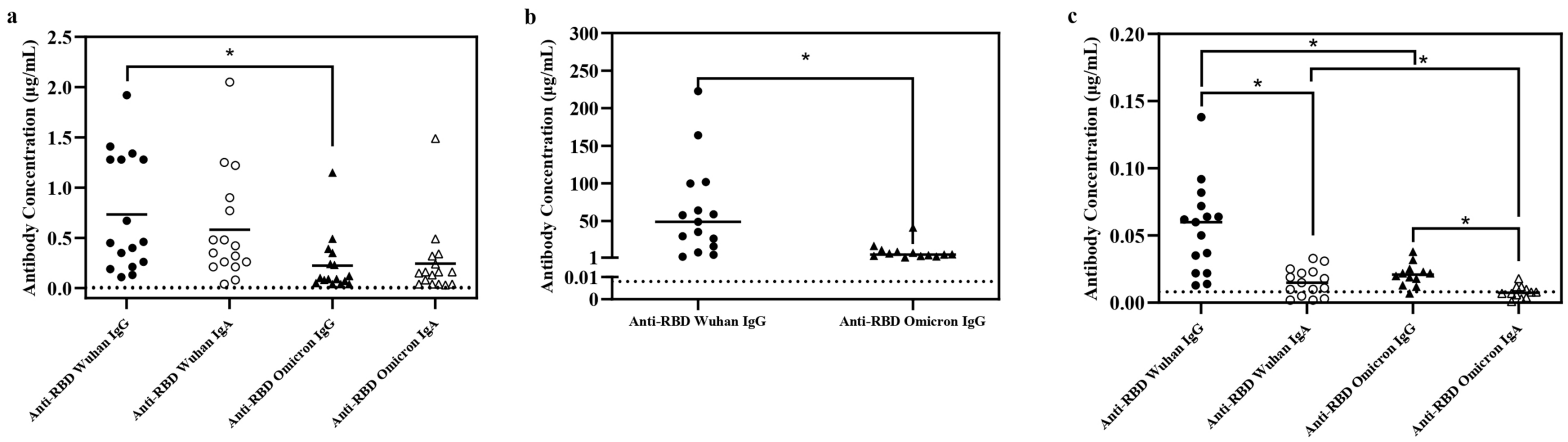
Antibody concentrations differ among tissue types. Anti-RBD antibodies (IgG and IgA) against the Wuhan and Omicron strains were detected in all human milk samples (milk combined from the left and right breast) from 16 participants **(a)**. IgG anti-RBD antibodies measured in dried blood spots from 15 of the participants were about 100-fold higher than in the milk **(b)**. Anti-RBD antibodies (IgG and IgA) concentrations in saliva were 1,600-fold lower than in blood **(c)**. Background is denoted by the dotted line. *p < 0.05.

Milk samples from all 16 participants were positive for IgG and IgA antibodies against both the Wuhan and Omicron variants of SARS-CoV-2 (Figure 1a), as all milk samples had calculated concentrations three times above the background control. The levels of antibodies in milk ranged from 0.03 to 2.05 μg/mL (Figure 1a). The concentrations of anti-Wuhan RBD IgG were significantly higher than those of anti-Omicron RBD IgG, with means of 0.73 μg/mL and 0.22 μg/mL, respectively (*t* = 3.14, *p* < 0.05). There were no significant differences between anti-Wuhan and anti-Omicron anti-RBD IgA concentrations, nor between anti-RBD IgG and IgA within the Wuhan or Omicron variants.

Blood samples from all 16 participants were positive for IgG (Figure 1b). The concentration of anti-RBD IgG in blood was about 100-fold higher than that in milk. Anti-Wuhan RBD IgG blood concentrations showed the highest variation among participants, ranging from 2.36 to 222.72 μg/mL, with an average value of 62.63 μg/mL. The concentrations of anti-Omicron RBD IgG in blood were significantly lower, with an average value of 7.88 μg/mL, nearly eight times less than the anti-Wuhan RBD IgG (Figure 1b: t = 3.36, *p* < 0.05).

The saliva samples had the lowest concentrations of anti-Wuhan RBD and anti-Omicron RBD IgG and IgA (Figure 1c). Concentrations of antibodies in saliva were 16 hundred-fold lower than antibody concentrations in blood. Twelve of the 16 participants showed no positivity for at least 1 of the 4 anti-RBD antibody classes measured in saliva. Anti-Omicron RBD IgA had the lowest positivity of 37.5% and a mean concentration of 0.01 μg/mL, significantly less than anti-Omicron RBD IgG (*t* = 3.17, *p* < 0.05) and anti-Wuhan RBD IgA (*t* = 3.00, *p* < 0.05). All participants were positive for anti-Wuhan RBD IgG in their saliva, with an average concentration of 0.06 μg/mL, significantly higher than anti-Wuhan RBD IgA (*t* = 4.39, *p* < 0.05) and anti-Omicron RBD IgG (*t* = 4.18, *p* < 0.05). There were no significant correlations between anti-Wuhan RBD IgG in saliva with either milk or blood (*r* = 0.01, and *r* = 0.06, respectively).

### No correlation between antibody levels in milk and time since COVID-19 infection or immunization

**Figure 2:**
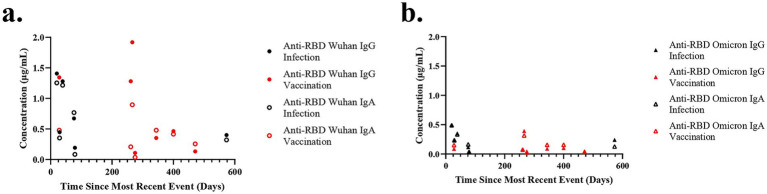
No correlation between antibody level and time since event. The time since the most recent event (infection or vaccination) was plotted against the anti-RBD Wuhan IgG and IgA antibodies **(a)** and anti-RBD Omicron IgG and IgA antibodies **(b)**. Five women reported having both a past COVID-19 infection and immunization. For these five, the antibody concentration for only the most recent event is plotted.

To determine the relationship between antibody levels and time since COVID-19 vaccination or infection, antibody concentration in milk was plotted against time elapsed since the most recent event (Figure 2). Five participants had both a prior COVID-19 infection and received a COVID-19 immunization, and for these five, only the most recent event is shown. Despite the wide range in time since the most recent event (20–573 days), there was no association with antibody concentration in milk for anti-RBD Wuhan (Figure 2a) or Omicron (Figure 2b) IgG or IgA. Interestingly, the milk of a woman 266 days past initial vaccination had the highest concentration of anti-RBD Wuhan IgG among participants with a past COVID-19 vaccination. The milk of another participant, only 79 days post-infection, had the lowest levels of antibodies detected among this cohort.

### No correlation between antibody levels in blood and time since COVID-19 infection or immunization

To determine the relationship between circulating levels of antibodies and time since COVID-19 vaccination or infection, antibody concentrations in blood were plotted against time since the most recent event (Supplementary Figure 2). There appeared to be a decrease in anti-RBD Wuhan IgG levels over time since vaccination (*r* = −0.75); however, the correlation was not significant. Similarly, correlations between time since recent event and antibody concentrations of anti-RBD Wuhan IgA and anti-RBD Omicron IgG and IgA were not significant, despite the wide range in time since the most recent event (20–573 days).

### Cytokines in milk

**Figure 3:**
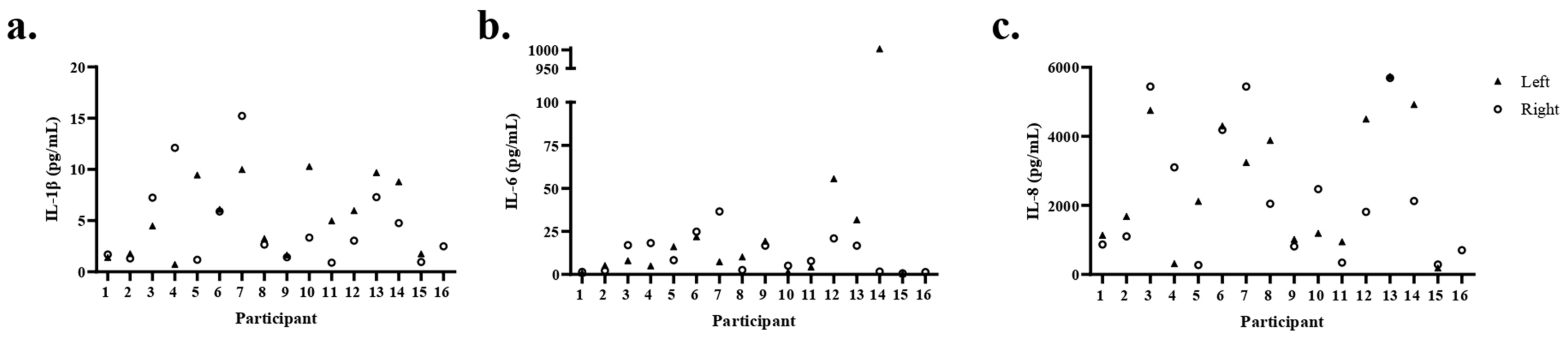
Cytokine concentrations in milk vary among participants and breasts. Concentrations of IL-1β **(a)**, IL-6 **(b)**, and IL-8 **(c)** were measured in milk from the left and right breasts of 16 participants. For participant 16, measurements were made in milk from only the right breast due to an insufficient sample.

Table 2 provides the descriptive statistics for the 10 cytokines comprising the inflammatory panel. While most analytes are detected in most samples, the CVs of technical replicates are above 10% for seven cytokines (INFγ, IL-10, IL-12p70, IL-13, IL-2, IL-4, and TNFα), ranging from 11.5% for TNFα to 37.58% for IL-4. Given the small sample size of this pilot study and the high CVs, we did not further analyze these seven cytokines. Three cytokines, IL-1β, IL-6, and IL-8, with mean CVs below 10%, were further analyzed. As shown in Figure 3, the concentrations of IL-1β, IL-6, and IL-8 were similar between breasts for most participants. However, there were some robust differences. For example, the concentration of IL-6 was 600-fold higher in the left than the right breast for participant 14.

**Table 2:** Cytokines in human milk.

Analyte	Average CV (%)	Left breast (*n* = 15*)					Right breast (*n* = 16)				
		Median Conc. (pg/mL)	SD	IQR	Range	Detection (%)	Median Conc. (pg/mL)	SD	IQR	Range	Detection (%)
IL-1β	9.82	5.48	3.6	1.77–9.13	1.41–10.29	100	3.04	4.2	1.4–6.23	0.9–15.22	100
IL-6	6.69	7.96	15.2	5.01–19.26	0.45–1,003	100	8.26	10.6	2.02–17.3	0.61–36.55	100
IL-8	7.97	2,681	1887	1,078–4,403	186.17–5,740	100	2050	1933	785.32–3,372	271.32–5,691	100
INF-γ	15.86	26.16	49.59	2.06–2.82	0.92–152.62	100	26.14	51.85	2.51–6.32	1.25–147.52	100
IL-10	19.66	1.79	3.46	0.29–0.95	0.12–11.99	100	2.65	5.48	0.26–0.89	0.12–18.01	100
IL-12p70	25.85	2.58	4.97	0.22–0.69	0.1–13.24	73.3	4.06	8.96	0.19–0.5	0.08–27.75	75
IL-13	11.94	53.86	33.7	31.93–62.34	15.73–139.47	100	54.20	44.18	27.56–57.19	19.47–191.84	100
IL-2	13.48	6.32	11.47	1.21–3.63	0.06–37.84	100	8.45	16.94	1.05–3.27	0.35–58.83	93.8
IL-4	37.85	0.33	0.54	0.03–0.42	0.01–1.75	80	0.38	0.72	0.04–0.12	0.02–2.19	87.5
TNF-*α*	11.50	8.28	8.24	2.4–9.82	0.8–29.95	100	10.14	15.1	2.43–11.16	0.97–54.23	100

*There was insufficient milk to assess cytokine concentrations in the milk form the left breast of one participant.

The relationship between cytokine concentration in milk and time since the most recent event of COVID-19 infection or vaccination was assessed (data not shown). There was a significant correlation between IL-1β and time elapsed since COVID-19 vaccination (*r* = 0.58, *p* < 0.05). There were no significant associations between IL-6 and IL-8 and time since vaccination. There were no associations between time since COVID-19 infection and the concentration of either IL-1β, IL-6, or IL-8.

### Mammary epithelium permeability

**Figure 4:**
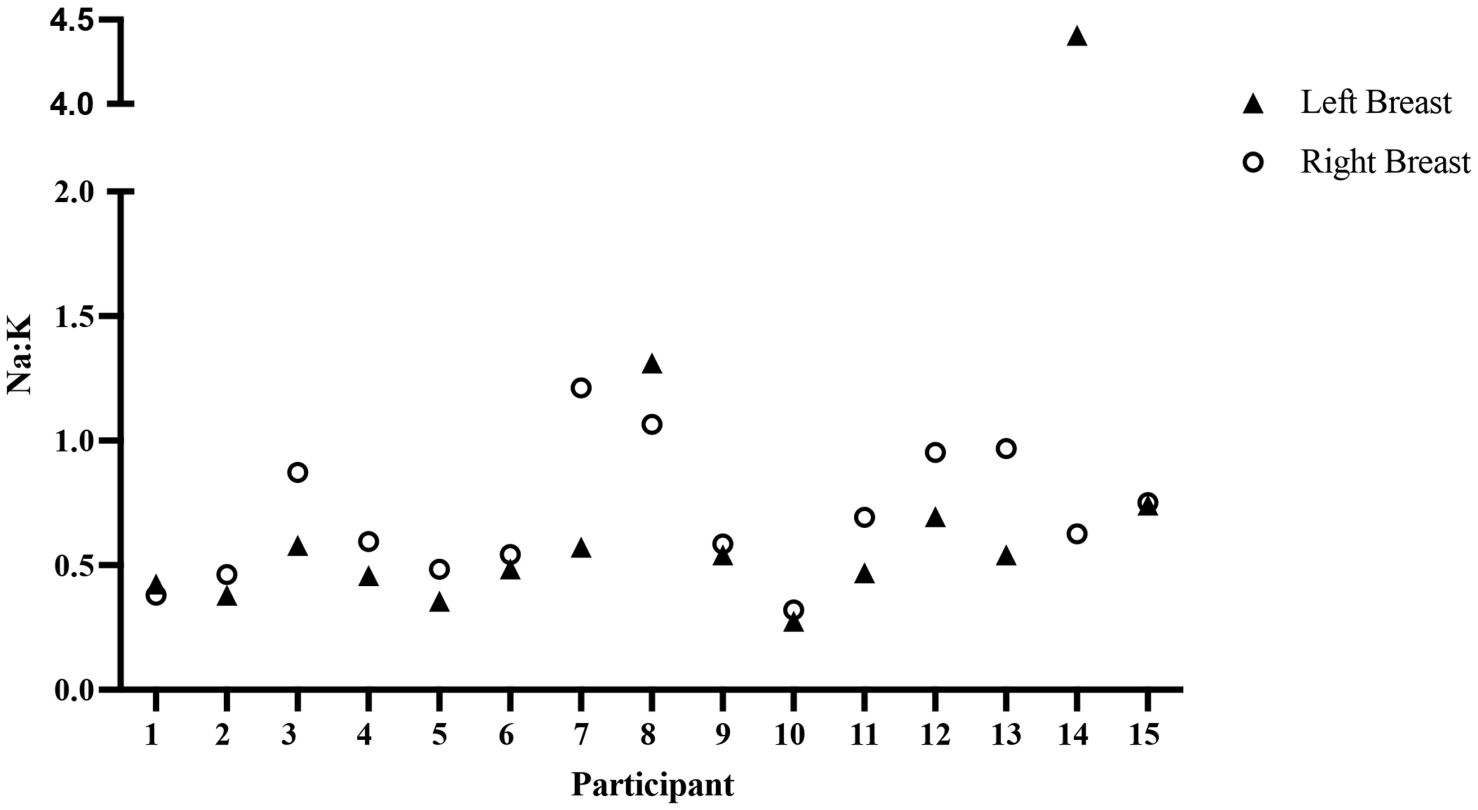
Mammary epithelium permeability varies little between breasts. Na: K, an indicator of mammary epithelium permeability, is shown for the left and right breasts of 15 participants. There was no available sample for participant 16.

Elevated mammary epithelium permeability during established lactation is considered a marker of inflammation and is frequently associated with higher levels of antibodies and cytokines (38–40). To assess mammary epithelium permeability, we determined the ratio of Na to K (Na: K) in milk. As shown in Figure 4, the Na: K values were similar between the left and right breasts for most participants. The most notable difference is seen for participant 14, who had a Na: K of 0.63 and 4.41 in the right and left milk samples, respectively (sevenfold difference). Participant 7 also had a substantially higher permeability in one breast (twofold difference). Of the 15 bilateral human milk samples measured, the Na concentrations ranged from 4.4 to 50.5 mmol/L, the K concentrations ranged from 10.11 to 22.4 mmol/L, and, overall, Na: K did not differ between the left and right breasts (*t* = −0.42, *p* > 0.05).

### No significant association between mammary epithelium permeability and antibody concentration in milk

**Figure 5:**
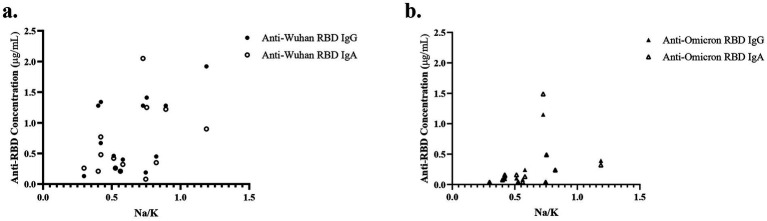
No association between mammary epithelium permeability and antibody concentration. The association between mean Na: K and anti-RBD Wuhan **(a)** and Omicron **(b)** antibody concentrations for 13 participants is shown. For each participant, antibody measurements were made in samples of milk combined from her left and right breasts. Na: K are the mean values of the measurements made separately in the left and right breasts. Two participants with highly different Na: K in their left and right breasts are excluded (participants 7 and 14), and no Na: K measurements were available for participant 16.

As the antibodies in milk were measured in a single combined sample (equal volumes from the left and right breast) for each participant, we used the following procedure to assess the association between mammary epithelium permeability and antibody concentration in milk. We removed data from the two participants whose permeability differed between breasts (participants 7 and 14), calculated the mean Na: K value for the remaining 14 participants, and compared this with the concentration of the two anti-RBD Wuhan antibodies (Figure 5a) and the two anti-RBD Omicron antibodies (Figure 5b). All four antibodies (anti-RBD Wuhan IgG and IgA and anti-RBD Omicron IgG and IgA) show an increase with permeability (*r* = 0.5, *r* = 0.42, *r* = 0.45, *r* = 0.33, respectively); however, none of the correlations are significant (*p* > 0.05).

### Significant association between mammary epithelium permeability and cytokine concentration in milk

**Figure 6:**
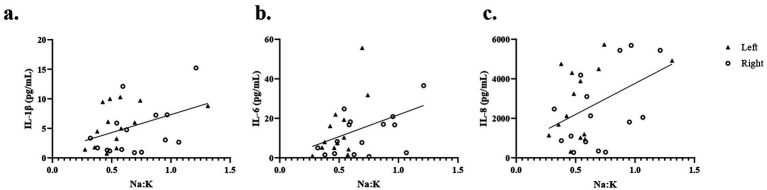
Associations between mammary epithelium permeability and pro-inflammatory cytokines in milk. Concentrations of IL-1β **(a)**, IL-6 **(b)**, and IL-8 **(c)** in milk are correlated with Na: K, a measure of permeability. Cytokines and permeability were measured in milk from the left and right breasts of all participants; note that data for the left breast of participant 14 were excluded for this analysis.

We next asked if mammary epithelium permeability was associated with the concentrations of IL-1β, IL-6, or IL-8 in milk. Since both cytokines and Na: K were measured in each breast, we included all values except the single outlier based on Na: K (the left breast of participant 14). As shown in Figure 6, there is a significant correlation between Na: K and IL-1β (Figure 6a), IL-6 (Figure 6b), and IL-8 (Figure 6c; *r* = 0.41, 0.40, 0.40, respectively, *p* < 0.05). This association is also clear at the individual level. Participants 7 and 14 had elevated Na: K in one breast (Figure 4) and had corresponding high cytokine levels in the same breast (Figure 3).

## Discussion

All women in this pilot study had detectable levels of antibodies against SARS-CoV-2, both IgG and IgA, in their milk, highlighting a major benefit of breastfeeding, the passive transfer of antibody protection to infants (2, 41). The protection appears to be long-lasting, as even milk collected more than a year after the most recent event (either COVID-19 infection or vaccination) had significant concentrations of anti-SARS-CoV-2 antibodies. This is in agreement with the literature showing that the secretory IgA response against SARS-CoV-2 in milk is highly durable (31, 42) and the increased concentration of immunoglobulins with prolonged breastfeeding (43). These findings support continued breastfeeding among all mothers, as both vaccinations and maternal infections provide protective antibodies to the breastfed infant.

Time since infection or immunization did not explain the levels of anti-SARS-CoV-2 antibodies in milk (Figure 2) or blood (Supplementary Figure 2). It is possible that the high levels of antibodies at later times points are due to unrecognized infections, but the low levels of antibodies close in time to an infection or vaccination represent variability in response. Similarly, we previously found significant individual variation in both initial antibody response and over time in both milk and blood (2, 4, 33).

One goal of this study was to include DBS and saliva samples to further our understanding of maternal protection (blood spots) and to assess the possibility of collecting infant saliva in lieu of a blood spot heel prick for future research. IgG is the predominant antibody class in blood, and increased levels have been detected up to a year after infection (36). All women in this study had blood levels of IgG antibodies against SARS-CoV-2 at concentrations about 100-fold higher than those in milk. Both IgA and IgG anti-SARS-CoV-2 antibodies have been detected in saliva (33, 35, 44). Our findings confirm this result, and as expected, the levels of antibodies in saliva were substantially lower than in blood (1600-fold) and milk. As the concentrations of anti-RBD Wuhan IgG were not correlated with those in milk or blood, it appears that saliva specimens may not yet be a reasonable fluid for analysis of immune responses to infections similar to COVID-19. More sensitive assays and/or different collection and concentration methods should be pursued, as analysis of saliva would provide a larger number of consenting participants in future studies.

Milk and blood had significantly lower levels of antibodies against the Omicron variant as compared to the original Wuhan virus. A lower response to the Omicron variant is expected, given that sample collection occurred before the updated Bivalent BA.4/BA.5 vaccine was available, although women may have had exposure to the Omicron variant through natural infection, as this variant was first detected in late 2021 and spread rapidly (45). Recent studies, however, suggest that the lower response to the Omicron variant may be due to immune imprinting, an immune response to an initial exposure that imprints itself on the immune system, reducing responses to subsequent infections and vaccinations (33, 46, 47). It is also possible that the use of the Wuhan-Hu-1 standard curve to calculate the concentration of antibodies against the Omicron variant contributed to the lower observed concentrations of anti-Omicron antibodies, as the binding efficiencies between the two strains may differ. However, we previously found that the binding efficiencies were similar between the Wuhan-Hu-1 and Omicron BA.4/BA.5 RBD (33).

Another goal of our research was to assess the relationship between the permeability of the mammary epithelium and concentrations of antibodies and cytokines in milk. In mature milk of women without mastitis, the permeability of the mammary epithelium is low due to closed paracellular pathways that restrict constituents in blood from passing into the lumen (48, 49). Multiple factors, such as infection, inflammation, milk accumulation, and stress, can disrupt the tight junctions that maintain the closed paracellular pathway, and thereby allow sodium (Na) ions to pass to the lumen (48, 50). A ratio of Na: K above 0.6 is often used to indicate elevated mammary epithelium permeability (37, 51, 52) and is frequently associated with subclinical mastitis. Elevated mammary epithelium is associated with multiple changes in milk composition, such as increased levels of cytokines (37). Cytokines in milk can have multiple consequences. They can facilitate the recruitment and activation of lymphocytes, promoting immunological tolerance and maturation of the infant’s immune system (30). Elevated cytokines in milk can also negatively affect the development of oral tolerance (53), leaving the infant more susceptible to developing food allergies, irritable bowel disease, and celiac disease (54).

The extent to which cytokines in milk are altered due to infection with or vaccination against SARS-CoV-2 remains unclear. We previously found that cytokines in milk were associated with the severity of COVID-19 symptoms (1) and that they increased in milk after mRNA vaccination (4), but we also did not see an increase in most cytokines in milk among a cohort of women with COVID-19 (55). Indeed, the present finding that cytokine concentrations were not correlated with anti-RBD antibodies is consistent with a lack of a cytokine response in milk to COVID-19. The concentrations of cytokines detected in milk in this study are within the range of cytokine concentrations measured in milk before COVID-19 (56). It is important to recognize that the concentrations of cytokines in milk vary greatly among individuals, between breasts, and over lactation duration, and all of the factors contributing to this wide range are not presently understood.

Our finding of a weak but significant correlation between levels of three proinflammatory cytokines IL-6, IL-8, and IL-1β (57, 58) and mammary gland permeability, as measured by Na: K (Figure 6), is consistent with the literature. For most participants, the Na: K ratios and cytokine concentrations were similar between milk obtained from both the left and right breasts; however, there were some notable exceptions that suggest increased permeability is an appropriate indication of increased cytokine transfer to infants. For example, the Na: K ratio in milk from the left breast of participant 14 was seven times greater than in the milk from her right breast, and concentrations of IL-1β, IL-6, and IL-8 were all increased in the left milk sample, with IL-6 being nearly 1,000 times higher in her left as compared to her right breast. Similarly, the Na: K and cytokines were higher in the milk from one breast as compared to the other for participant 7. However, the relationship between mammary gland permeability and cytokines in milk is not straightforward. Participant 13 had the highest levels of IL-8 in milk from both her left and right breasts, but had a low Na: K ratio, and low to moderate levels of IL-1β and IL-6. Interestingly, no woman reported mastitis.

There are several limitations to this pilot study. First, the study included only 16 participants who were recruited around a single breastfeeding conference and,therefore, may not be representative of a larger population. Second, concentrations of the anti-RBD antibodies against the Omicron strain were calculated with a standard curve for the Wuhan-Hu-1 strain. Third, the cytokine analysis was restricted to a single inflammatory panel, and several of the analytes measured in technical replicates had high CVs. Fourth, control milk samples collected before COVID-19 were not included.

## Conclusion

The immune response present in human milk after systemic infection or vaccination varies widely among individuals, with potential consequences for the health of the breastfeeding infant. Understanding the mechanisms underlying this diverse response is important and could lead to improved infant health. The variability in response highlights the need to ensure that studies of human milk include diverse populations, collect and analyze milk from both breasts, and consider mammary gland permeability as a mitigating factor. Recruitment centered around community events important to targeted groups or community members may be an effective approach for diversifying participants in health-related studies.

## Data Availability

The raw data supporting the conclusions of this article will be made available by the authors, without undue reservation.

## Supplementary material

SUPPLEMENTARY FIGURE 1 Image of the recruitment flyer.

SUPPLEMENTARY FIGURE 2 The levels of anti-RBD IgG detected in blood were plotted against time since most recent event of either COVID-19 infection or vaccination. There was no significant correlation between time since the most recent event and antibody concentration in blood.
