# Supplementary material for: Immune response in breastmilk of Black women to SARS-CoV-2 infection and vaccination against COVID-19
Source: Front Nutr. 2026 Jan 12;12:1703784. doi: 10.3389/fnut.2025.1703784 (PMC12833877; doi:10.3389/fnut.2025.1703784)
Supplement: SUPPLEMENTARY FIGURE 1 — Image of the recruitment flyer. [file Data_Sheet_1.PDF]

Black Moms We Need You!

We're looking for Black Breastfeeding Moms to participate in a health study of the special benefits of breastmilk during COVID-19.

Breastfeeding moms see how your milk protects your baby against COVID-19

- You will receive $100 for participating.
- Drop off your milk sample at the ROSE - Black Breastfeeding & Birth Justice Summit August 25 – 27, 2022 at the New Orleans Marriott Warehouse Arts District

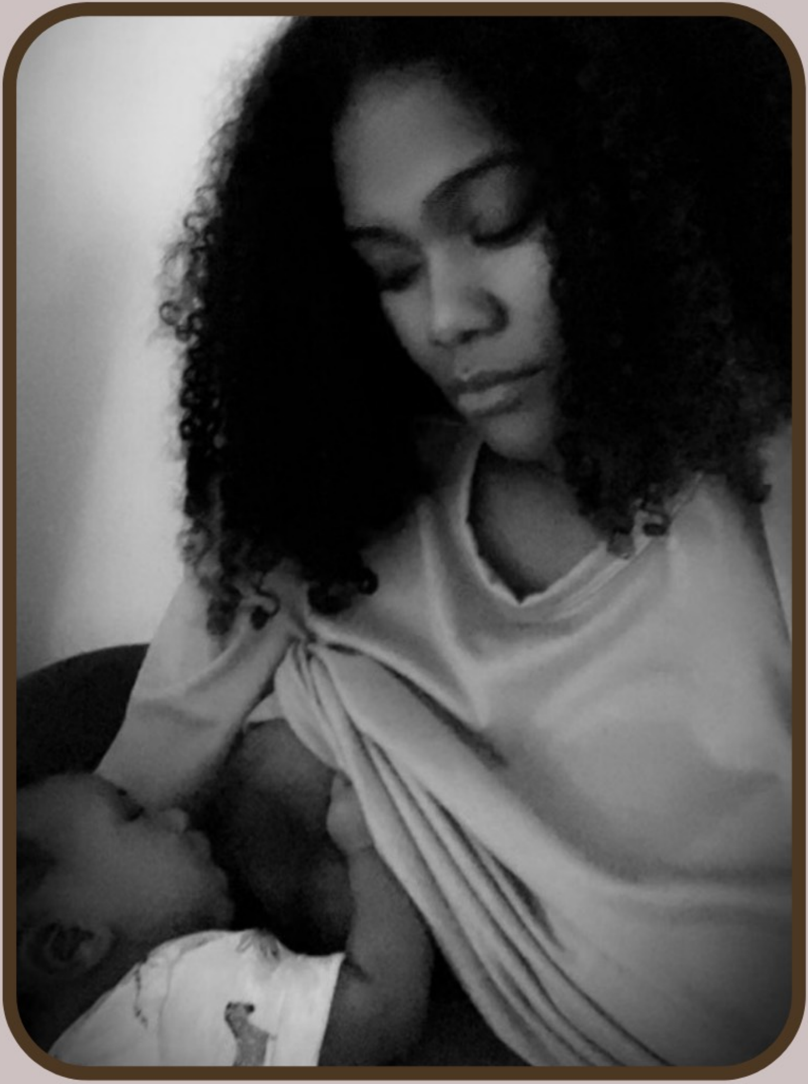

Register Here

For more information contact Halle Neeley
halleneeley@breastfeedingrose.org

ROSE
Reaching Our Sisters Everywhere

University of Massachusetts Amherst
