# Supplementary figures and images for: Immune response in breastmilk of Black women to SARS-CoV-2 infection and vaccination against COVID-19
Source: Front Nutr. 2026 Jan 12;12:1703784. doi: 10.3389/fnut.2025.1703784 (PMC12833877; doi:10.3389/fnut.2025.1703784)

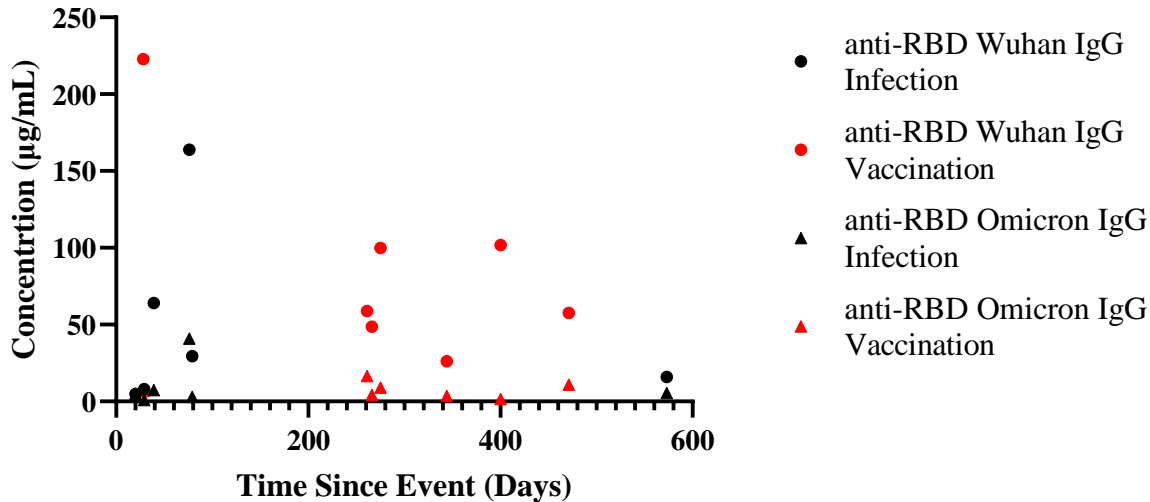

Supplement: SUPPLEMENTARY FIGURE 2 — The levels of anti-RBD IgG detected in blood were plotted against time since most recent event of either COVID-19 infection or vaccination. There was no significant correlation between time since the most recent event and antibody concentration in blood. [file Data_Sheet_2.PDF]
